# Quantifying the potential market for new contraceptive technologies: global projections of 2040 contraceptive needs and preferences

**DOI:** 10.12688/gatesopenres.13400.1

**Published:** 2021-10-22

**Authors:** Michelle Weinberger, Meghan Reidy, William Winfrey

**Affiliations:** 1 Avenir Health, Takoma Park, MD, 20912, USA; 2 Avenir Health, Glastonbury, CT, 06033, USA

**Keywords:** Contraception, family planning, user preferences, market segmentation

## Abstract

**Background:** Despite a wide range of contraceptive methods, unmet need persists. New contraceptive technologies (CTs) have the potential to improve uptake and continuation. CT development has a long-time horizon; products will be introduced into markets that look very different than today. Identifying viable investments requires an understanding of these future markets. For this work the 2040 potential contraceptive market is described utilizing seven market segments based on marital status, fertility preferences, and patterns of sexual activity outside of marriage.

**Methods:** Market size estimates are developed by country for all countries in the world for a current market (2020) and a future market (2040). United Nation’s (UN) population projections of the number of women of reproductive age (WRA) form the basis of this work. WRA are then segmented into market segments based on marital status, fertility intentions, and patterns of sexual activity outside of marriage.  Each segment is further subdivided by contraceptive use versus non-use.  Segmentation draws from UN projections, household surveys, census data, and modeling techniques developed for this work.

**Results:** The largest market increases will be seen in Africa; most notably among the segment of married women wanting no more children. By contrast, Asia will see declines across all three married segments, coupled with increases among sexually active unmarried segments.  Levels of contraceptive use are projected to vary widely by segment, with differential patters across regions.

**Conclusions:** This analysis projects the impact of demographic changes, evolving fertility preferences, shifts in sexual activity outside of marriage and increased utilization of contraceptives in shaping the contraceptive market of 2040. Results show that there is not one global market, but distinct markets that vary in size and shape across the world. This diversity suggests that a range of different new CTs could have potential for uptake.

## Introduction

Despite the existence of a wide range of contraceptive methods, more than 200 million women across developing countries who do not want to become pregnant are not using modern contraceptives ( Guttmacher, 2017). In addition, among women who use contraception, dissatisfaction with methods including experience of side effects and other method attributes can lead to inconsistent use or discontinuation, thus putting women at greater risk of unintended pregnancy ( Institute of Medicine, 2004; Jain & Winfrey, 2017). 

The development of new and innovative contraceptive technologies (CTs) has been recognized as a potential solution to improving contraceptive uptake and continuation ( Darroch *et al.*, 2011; FHI360, 2017; Institute of Medicine, 2004). Large global initiatives are making investments in developing new technologies (for example, the BMGF CTI Exchange and the USAID-funded Envison FP project ^1^). Investments in new technologies are being made at different stages in the development pipeline—ranging from early discovery to clinical trials ( Calliope, 2020). 

Products in the early discovery stage or not yet in the development pipeline would likely not be available at scale until at least 2040. This long-time horizon means that the markets into which these products will be introduced will likely look much different than today, with user needs and preferences evolving in the face of demographic changes coupled with changes in marriage patterns, sexual activity and fertility preferences. Identifying viable investments with relevant product features requires an understanding the world of 2040 into which a new CT would be introduced.

For this work, the 2040 potential contraceptive market is described utilizing seven market segments based on marital status, fertility preferences, and patterns of sexual activity outside of marriage. These characteristics were selected because they are meaningfully related to contraceptive needs and preferences and can be estimated and projected across countries. The distribution of women of reproductive age (WRA) across these seven segments were estimated based on current patterns (defined as 2020) and projected to 2040 utilizing a range of existing data sources and new modeling methodologies. Within each segment, women are further segmented by contraceptive use to allow for quantifying two distinct pathways for new CT utilization: first, women who would likely be using one of the currently available methods could switch to a new CT; second, women who would likely not be using one of the currently available methods might adopt a new CT. 

This work builds on a solid base by capitalizing on sophisticated statistical projections developed by the United Nation’s Population Division (UNPD) ( United Nations, 2019a; United Nations, 2019b). This is not the first effort that draws from these projections or seeks to project future contraceptive use and needs in ways that go beyond the UNPD projections. The Reproductive Health Supplies Coalition (RHSC) Commodity Gap Analysis projects contraceptive use and costs to 2030 for low-and-middle-income countries, accounting for shifts in method mix as well as overall changes in contraceptive use ( Weinberger *et al.*, 2019). Work by Biddlecom and colleagues projected scenarios for contraceptive use, costs, and impacts among adolescents in developing countries to 2030 ( Biddlecom *et al.*, 2018). The work presented in this paper takes a new and unique approach by projecting segments aligned to future contraceptive needs and preferences. It also takes a fully global scope, looking at all countries of the world in order to assess both commercial and subsidized markets for new CTs. Finally, in order to align with the time horizons of new product development, this work also looks further into the future, projecting changes to 2040. 

The market projections described in this paper serve as a basis for two subsequent modeling efforts. First, the uptake of a new CT is estimated by applying different likelihoods to switch to or adopt a new CT based on method attributes and preferences of women in the market segment. This is informed, in part, by results from a large-scale online discrete choice experiment ^2^. Second, the health impact of this uptake is quantified in terms of unintended pregnancies averted based on method effectiveness and switching and adoption patterns. Continued work in this area helps inform decisions about how to fund innovations in contraceptive technology. 

## Methods

A set of seven mutually exclusive and exhaustive market segments were defined as follows:

1. Married women who want another child soon (within 2 years; referred to as ‘soon’)
2. Married women who want another child later (2+ years; referred to as ‘space’)
3. Married women who want no more children (referred to as ‘limit’)
4. Never-married women who have had recent sex (within 30 days; referred to as ‘recent sex’)
5. Never-married women who have had sex but not recently (30+ days; referred to as ‘non-recent sex’)
6. Never-married women who have never had sex (referred to as ‘no sex’)
7. Formerly married women

Segments needed to be based on characteristics that are likely to relate to women’s contraceptive needs and preferences. These characteristics needed to be queried in many surveys, especially for countries where rapid economic and social change are likely to occur over the next 20 years. The characteristics found to be both readily available and most salient were fertility preferences among married women and frequency of sexual activity amongst never-married women.

Within each segment women were disaggregated into users and non-users based on use of any contraceptive (including traditional methods). Further work was done to estimate method mix within each segment; these results are not covered in this paper. Method mix estimates are used to apply different probabilities of switching to a new CT in subsequent modelling. 

Estimates were developed by country for 2020 and projected to 2040 for the 201 countries included within the World Population Prospects 2019 (WPP2019) ( United Nations, 2019a). The median number of WRA in 2020 and 2040 was used. Next, total WRA were segmented by marital status using UNPD marital status projections ( United Nations, 2017). This segments women into those who are currently married or in union versus unmarried (including never-married and formerly married). Throughout this paper ‘married women’ is used for simplicity, but in fact this refers to married or in union women. As the UNPD marital projections end in 2030, a linear trend was created to project forward to 2040. Further segmentation is handled differently for married women and unmarried women as described below.

Many input indicators were calculated by cross-tabulating data from the Demographic and Health Survey (DHS) ( ICF, 2004-2019) and Multiple Indicator Cluster Survey (MICS) ( UNICEF, 2019) using STATA SE12. These analyses weighted results using the standard weight variables provided in DHS and MICS datasets and dropped results where the denominator was less than 25. Details on indicators used are provided in the relevant sections below. Analysis was done on the most recently available survey in each country unless otherwise specified. 

The size and distribution of the segments are the main result of this paper. As such, some intermediary results required to create the segments are presented within the methods section below.

### Married women

***Married women by segment.*** Fertility preference distributions were taken from survey data that asks women about their desire to have more children. Surveys allow for a wider range of responses than the three segments being considered for this work. To address this, women who want more children but were unsure of timing were grouped in with those who want after 2+ years (spacers), and women who were undecided overall about having a(nother) child, women who they (or their partner) are already sterilized, and women who are declared infecund were grouped into married women wanting no more children (limiters).

In order to estimate and project the distribution of married women into the three segments, linear regressions were run on DHS survey data to quantify relationships between the desire to space versus the total fertility rate (TFR) and the desire to limit versus TFR respectively. In order to capture trends over time, analysis included all available surveys, not just the most recent. In total 280 surveys from 86 countries spanning 1985 to 2018 were included based on results obtained from StatCompiler (IFC, 2012) (see Data availability section). The share of married women falling into each of the three segments was calculated using the definitions described above.

**Figure 1.**
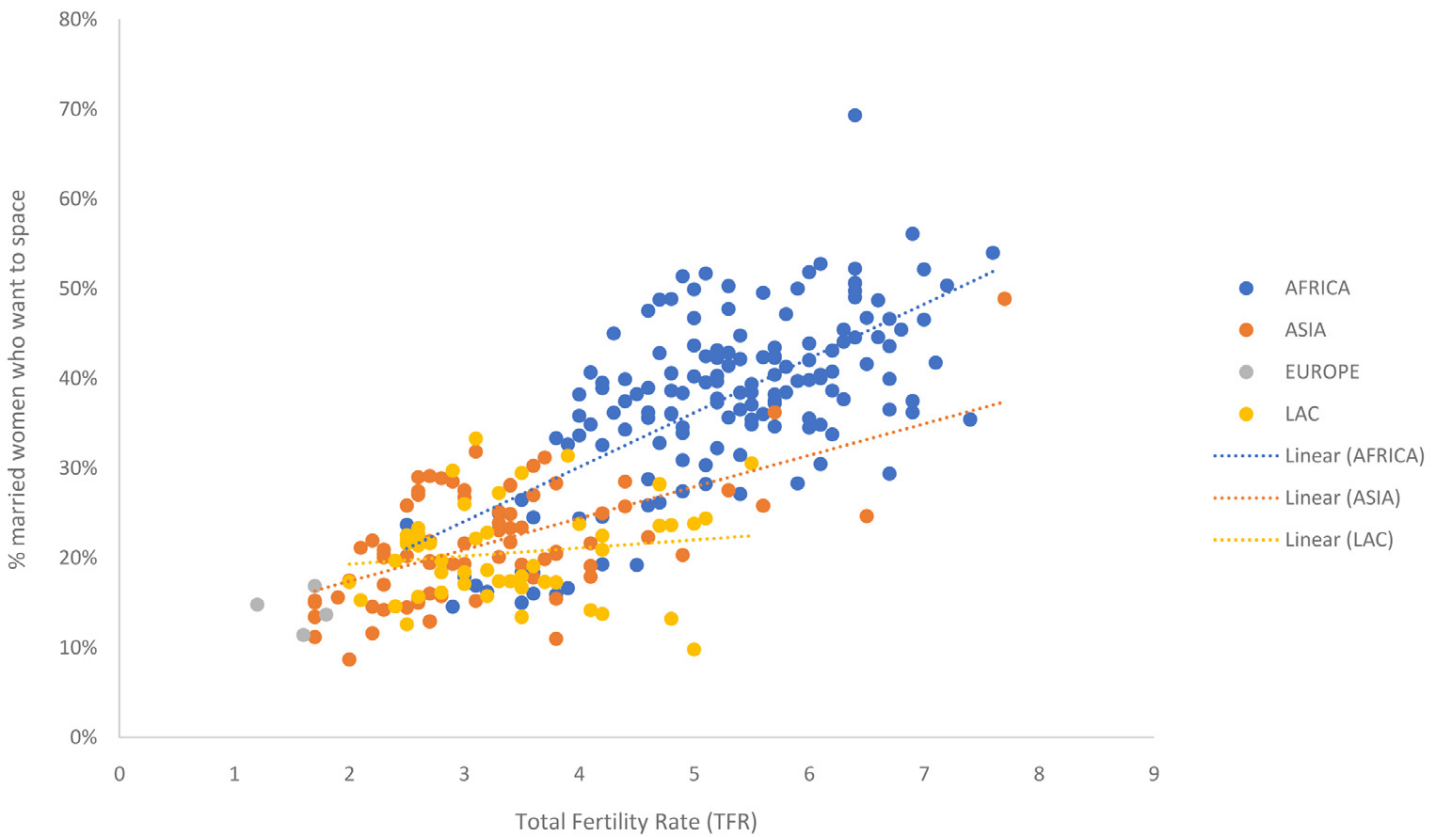
Relationship between TFR and share of married women who want to space their next birth. The dots in the scatterplot represent survey data from all available Demographic and Health Surveys (DHS), color coded by region. The data shows the relationship between the total fertility rate (TFR) and the share of married women who want to space their next birth.

**Figure 2.**
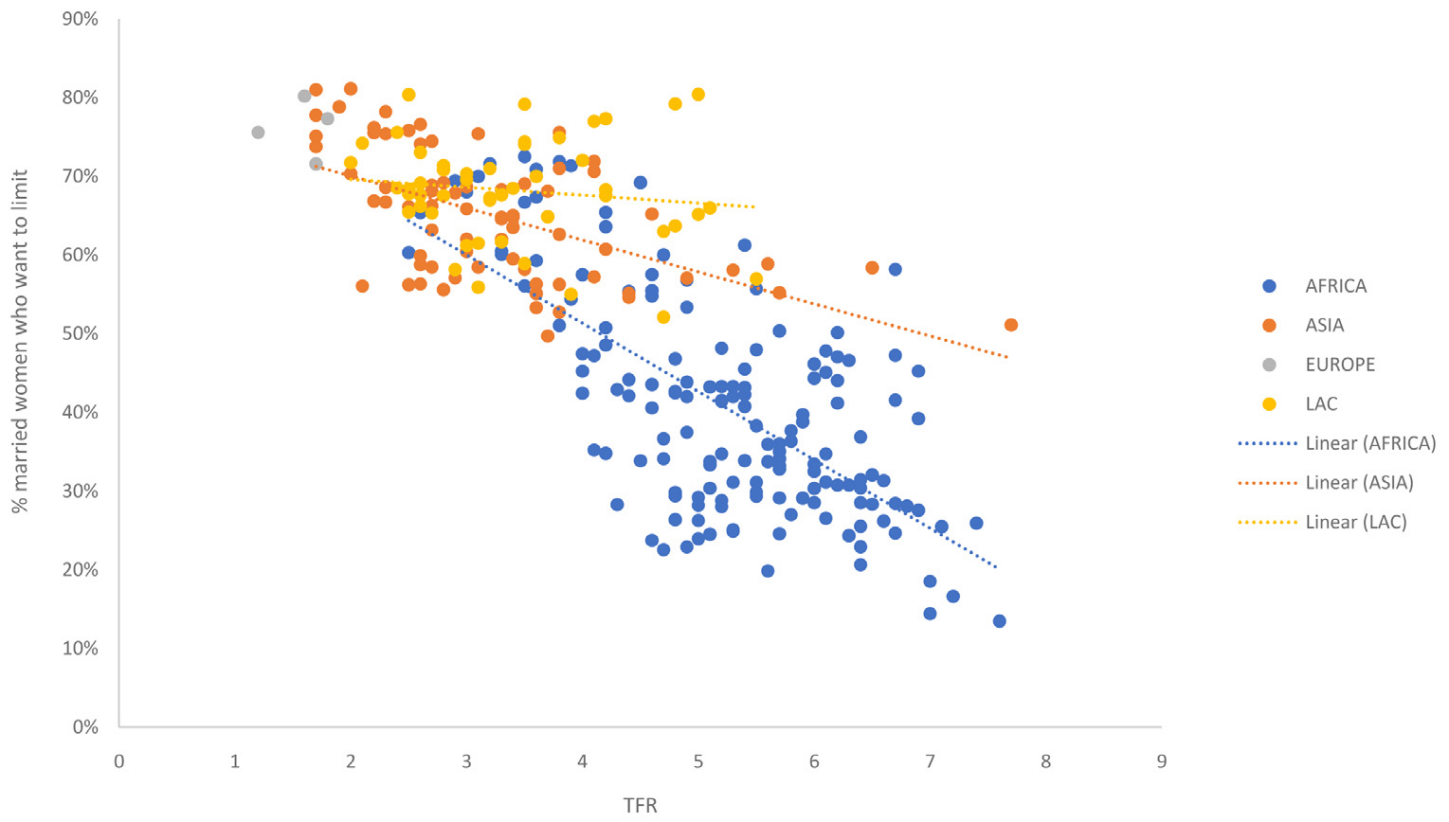
Relationship between TFR and share of married women who want no more children (limiters). The dots in the scatterplot represent survey data from all available Demographic and Health Surveys (DHS), color coded by region. The data shows the relationship between the total fertility rate (TFR) and the share of married women want no more children (limiters).

Two separate linear regressions were created; one relating TFR to married spacers ( Figure 1) and one relating TFR to married limiters ( Figure 2). While there appears to be some regional differentiation in these patterns, this is largely driven by availability of data (e.g., limited coverage of survey data from Asian countries with high TFRs and limited coverage of survey data from Africa in countries with low TFRs). Therefore, it was decided to create a single global regression, rather than regional regression, to quantify the relationship between TFR fertility preferences. A global ‘convergence point’ was calculated for each segment as the expected value when replacement TFR (2.1) is reached; giving the expected share of WRA who will be in each segment when TFR reaches replacement ( Table 1).

**Table 1.** Global regression results for spacers and limiters.

	Spacers	Limiters
Intercept	2.6%	93.4%
Slope	6.4%	-9.5%
R ^2^	62.3%	62.6%
Convergence Point	16%	74%

This table shows the results from of global regressions from Figure 1 and Figure 2, including the intercept, slop, and coefficient of determination (R ^2^). The convergence point represents the share of married women classified as spacers and limiters when the total fertility rate (TFR) reaches replacement levels (2.1).

For countries with survey data, we assumed a linear progression from the most recent survey data point to this global convergence point; the slope and intercept of this line were calculated for each country. This was done separately to quantify the relationship between married spacers and TFR and married limiters and TFR. These were then used to calculate the share of women in these two segments from the estimated TFR in 2020 and the projected TFR in 2040 based on WPP2019. However, rather than using this calculated value, values from the most recent survey were used in three situations:

(1) TFR in the survey is at or below 2.3 (to capture countries already at or near replacement fertility)
(2) The share of married spacers < predicted value
(3) The share of married limiters > predicted value

For the remaining countries with no survey data, the slope and intercept from the global regression were used to calculate the share of women in each segment. Finally, for all countries the married soon proportion was calculated as the residual, as the three segments must sum to 100%.

***Married contraceptive use.*** The overall contraceptive prevalence rate (CPR) for married women in each country is based on the median projections from UNPD Model-Based Estimates ( United Nations, 2019b). A curve fit to global data was used to extend projections from 2030 to 2040 accounting for plateauing of CPR. This curve is based on previous analysis estimating average growth rates at different levels of CPR ^3^. Once CPR reached 70% it was held constant going forward. For both the 2020 estimate and 2040 projection, CPR was then multiplied by the number of married WRA in the corresponding year to estimate total married users.

**Figure 3.**
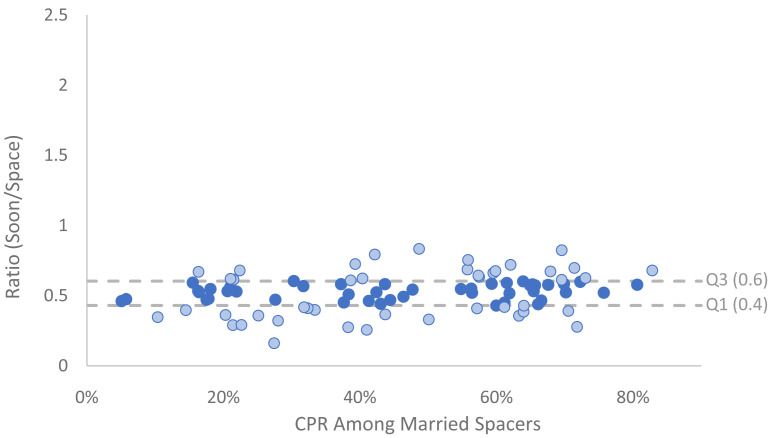
Ratio of married CPR: soon/space by CPR among married spaces. This graph shows data from recent surveys, showing the contraceptive prevalence rate (CPR) among married spacers compared to the ratio of CPR between women who want a child soon and those who wish to space. The dotted lines indicate the interquartile range (IQR). Light blue dots are outside of the range.

**Figure 4.**
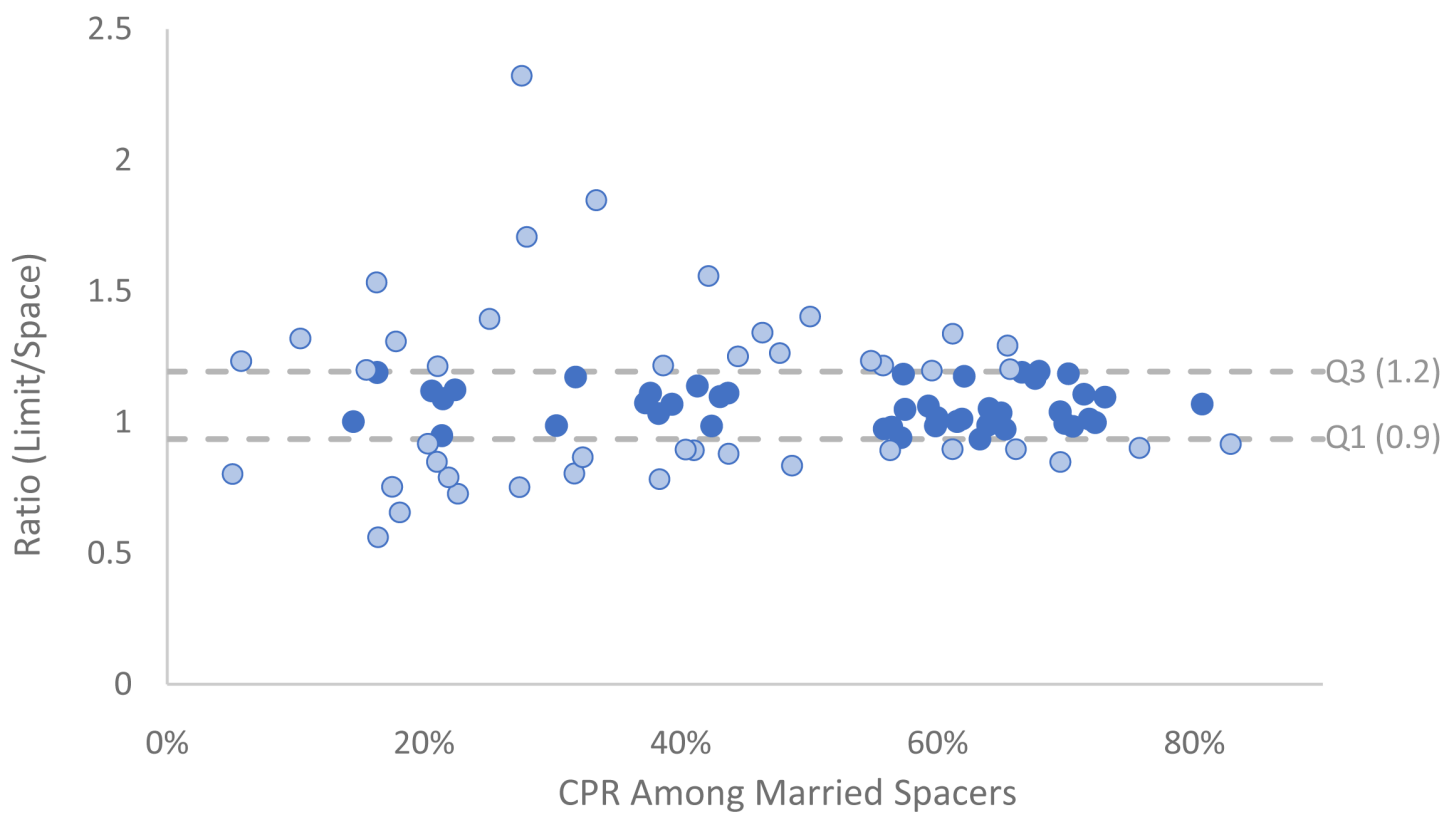
Ratio of married CPR: limit/space by CPR among married spaces. This graph shows data from recent surveys, showing the contraceptive prevalence rate (CPR) among married spacers compared to the ratio of CPR between women who want no more children (limiters) and those who wish to space. The dotted lines indicate the interquartile range (IQR). Light blue dots are outside of the range.

Next, the CPR in each of the three segments was calculated based on secondary analysis of the most recent DHS or MICS for 89 countries. The CPR for each married segment was calculated from the survey datasets. Next, two ratios were calculated: first the CPR of married soon divided by CPR of married spacers ( Figure 3), then CPR of married limiters divided by CPR of married spacers ( Figure 4). In all countries the CPR among women who want a child soon is lower than among married spacers (ratio < 1); with an average ratio of .52 ( Figure 3). The ratio appears to be fairly consistent across different levels of CPR.

There is more diversity in the relationship between the CPR of married limiters and married spacers ( Figure 4) ^4^. In 60% of countries the CPR for limiters is greater than the CPR for spacers; the average ratio for these countries is 1.22. The average ratio among countries where CPR is lower among limiters than spacers is .88. Across all data the average is 1.08. The ratio appears to be static across different levels of CPR and as CPR increases there is a tighter convergence in the data. 

These ratios were used to estimate CPRs for each segment that reconcile with the overall prevalence for the country in both 2020 and 2040, weighted by the number of married women in each segment. Thus, the estimates account for the shift in distribution of women across segments as described above. Where survey data was not available regional averages were used.

For the 2040 projection, if a ratio fell below the lower quartile (Q1) among all available survey data it was increased to Q1, if it fell above the upper quartile (Q3) among all available survey data it was lowered to Q3, otherwise the ratio was maintained. The CPR for the other two segments were then calculated by multiplying the CPR for married spacers by the corresponding ratio. By restricting 2040 ratios to be within the interquartile range (IQR), a comparable rule was applied across all countries that limits the skew of outliers. Survey estimates outside of the IQR are indicated with light blue on the graph the two figures above.

### Unmarried women

For unmarried women the split between formerly and never-married in 2020 was informed by DHS, MICS, national surveys, and data published in the UNPD World Marriage Data database ( United Nations, 2017). Estimates were available for 144 countries; for the remaining countries regional averages were used. For the 2040 projection the share of unmarried women who are formerly married was held constant.

Never-married women were then distributed among the three segments related to sexual activity: recent sex, has had sex but not recently (‘non-recent sex’), and has never had sex (‘no sex’). Recency of sex is meant to be a proxy for frequency of sex since most surveys only ask the former and not the latter. Given analysis is done at a population level and not on individuals, cross-sectional data on recency of sex will correlate highly with frequency. It is assumed that women having infrequent sex will have different contraceptive needs and preferences than those having more frequent sex. The distribution of never-married women across the three segments were estimated through secondary analysis of DHS, MICS, and national surveys. The latest survey in each country was used for the 2020 estimate; regional averages were used for countries with no data.

Analysis of patterns of sexual activity among never-married women was conducted using data from DHS and MICS surveys from 76 countries, as well as estimates constructed from multiple data sources for the US and UK (NATSAL, CPS, and NSFG). Countries were assigned to categories based on the share of never-married women who have had sex, ranging from: low (<10%), low-med (10-29%), med-high (30-49%), high (50-70%), very high (>70%). An unweighted average profile was calculated using data for countries in each category ( Table 2). A general pattern is found that as the share of never married women who have had sex increases, so too does the share who had sex recently.

**Table 2.** Profiles of never-married sexual activity by category.

	Sexual Activity Among Never- Married Women		Recency of Sex (among those who have had sex)		
	Never Had Sex	Ever Had Sex	Not Recent	Recent	# Countries
Low (<10%)	97%	3%	73%	27%	14
Med-low (10–29%)	79%	21%	69%	31%	14
Med-high (30–49%)	60%	40%	65%	35%	22
High (50–70%)	42%	58%	64%	36%	19
Very high (>70%)	22%	78%	46%	54%	9

This table shows the average share of never married that have ever had sex and recency of sex for each of the five categories developed based on level of sexual activity. The final column indicates how many countries contribute data to each category. Across the categories the share of women who have never had sex declines, while the share that is recent increases.

Countries were then assigned to a 2040 category based on moving each country a step up from their current level (except those already in the very high category). For example, countries that were in the low category in 2020 were assigned to the medium-low category in 2040. A country was then given the average profile of their new category in 2040, with the exception of countries already in the very high classification whose most recent survey or regional average was above the average. In this case, the survey data or regional average was used for the 2040 projection.

**Figure 5.**
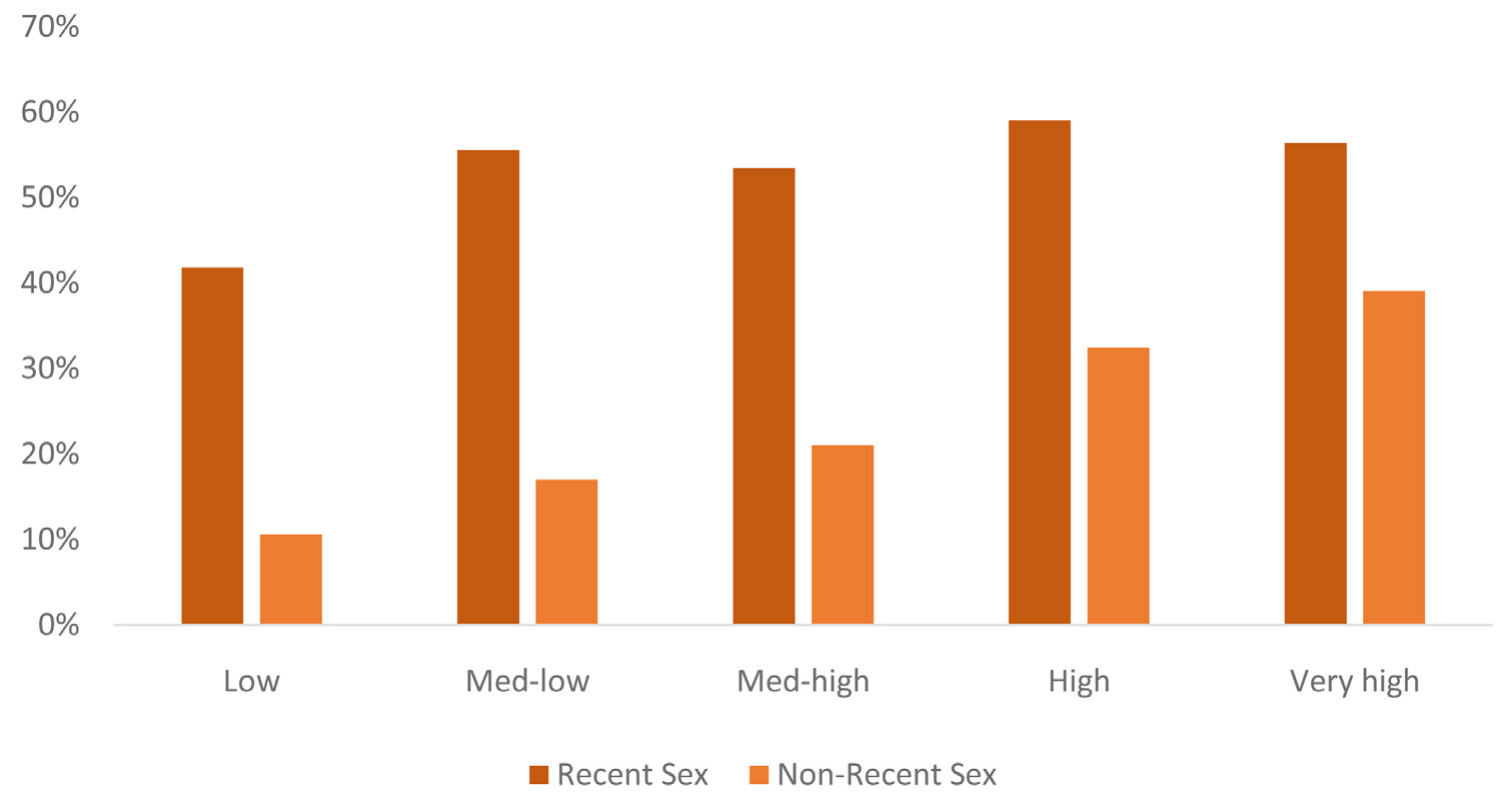
Recent sex and non-recent sex CPR by never-married category. This figure shows the average contraceptive prevalence rate (CPR) for never-married women who have had recent sex and non-recent sex for each of the five categories developed based on level of sexual activity.

The 2020 CPR for each unmarried women segment came from secondary analysis of survey data; or a regional average where no survey data was available. Data from DHS and MICS surveys from 71 countries contributed to the analysis of CPR by segment. The CPR for each group was based on the unweighted average of countries falling into the category (based on the share of never-married women who have had sex). Similar levels of CPR were found among never-married women who had sex recently across all categories, although with slightly lower levels in the ‘low’ category ( Figure 5). For never-married women who have had sex but not recently, CPR is always lower but increases across the categories.

The 2040 projections of CPR for each segment were based on the level of the shifted premarital sex categorization described above (taking the average CPR in each segment based on available survey data), unless the current CPR in the segment was already higher than that of the category.

## Results

Globally, the number of women of reproductive age is projected to increase from 1.9 billion to 2.2 billion. This growth will be largely concentrated in Africa, where the number of WRA is projected to more than double over this time period. By contrast, in some regions the number of WRA is projected to decline. When segmenting by marital status ( Table 3), different patterns of change begin to emerge as will be explored in the results below. 

**Table 3.** WRA by marital status and region in 2020 and 2040, in millions.

	Married			Unmarried		
	2020	2040	*Change*	2020	2040	*Change*
North, South & East Africa	110.0	154.1	*44.1*	79.4	133.	*53.6*
Western & Middle Africa	87.4	133.5	*46.1*	47.9	104.9	*57.1*
Southern Asia	367.3	379.8	*12.5*	138.7	186.9	*48.2*
East, Southeast Asia & Pacific	401.5	317.7	*-83.7*	177.8	193.5	*15.6*
West & Central Asia	54.9	59.1	*4.2*	34.3	48.1	*13.8*
Europe	90.4	71.0	*-19.4*	73.7	69.8	*-3.8*
Americas	138.6	142.9	*4.3*	118.5	123.0	*4.5*
**Total**	**1,250**	**1,258**	***8.1***	**670.2**	**859.2**	***189.0***

This table shows the number of women of reproductive age (WRA) in millions in 2020 and 2040, segmented by marital status and region. While overall the number of both married and unmarried women is projected to increase, some regions are projected to see declines.

### Married Women

***Married women by segment.*** The share of women of reproductive age who are married or in union is projected to decline from 65% in 2020 to 59% in 2040. A similar magnitude of decline is projected across most regions, with the exception the Americas, where marriage rates are projected to remain relatively unchanged. Despite a decline in the proportion of WRA who are married, in absolute terms the number of married women is projected to increase globally by 8.1 million, from 1.250 billion to 1.258 billion ( Table 3).

This global increase hides large changes by market segment. The number of married women who want a child soon and who want to space are both projected to decline (-6 million and -9.3 million), while the number of married women wanting to limit is projected to increase (+23.5 million).

The global changes described above are relatively minor when compared to the current number of women in each segment; these changes represent less than a 5% change in the number of women in each segment. However, the global figures hide large regional diversity. For example, in Western & Middle Africa increases are projected in all three groups, though the increase among married women limiters is substantially larger in both absolute and relative terms (+38.1 million, +116%). This contrasts with East & Southeast Asia and the Pacific where all three segments are projected to decline; the largest absolute decline is among married limiters, though the relative declines in the other two segments are similar (20% to 25%). See Table 4 for full details of change by region.

**Table 4.** Change in married women from 2020 to 2040 by market segment and region [abs change in millions (% change)].

	Married Soon	Married Space	Married Limit
**North, South & East Africa**	2.7 (15%)	2.9 (9%)	38.6 (63%)
**Western & Middle Africa**	3.1 (14%)	4.9 (15%)	38.1 (116%)
**Southern Asia**	-1.4 (-3%)	0.9 (2%)	13.0 (5%)
**East & Southeast Asia & Pacific**	-9.2 (-23%)	-14.9 (-25%)	-59.7 (-20%)
**Western & Central Asia**	-0.2 (-2%)	-0.3 (-3%)	4.7 (12%)
**Europe**	-1.7 (-20%)	-2.3 (-19%)	-15.5 (-22%)
**Americas**	0.6 (4%)	-0.6 (-3%)	4.3 (4%)
**Total**	**-6.0 (-4%)**	**-9.4 (-4%)**	**23.5 (3%)**

This table shows the change in the number of married women in each segment in millions between 2020 and 2040. The number in parentheses indicates the percent change.

**Figure 6.**
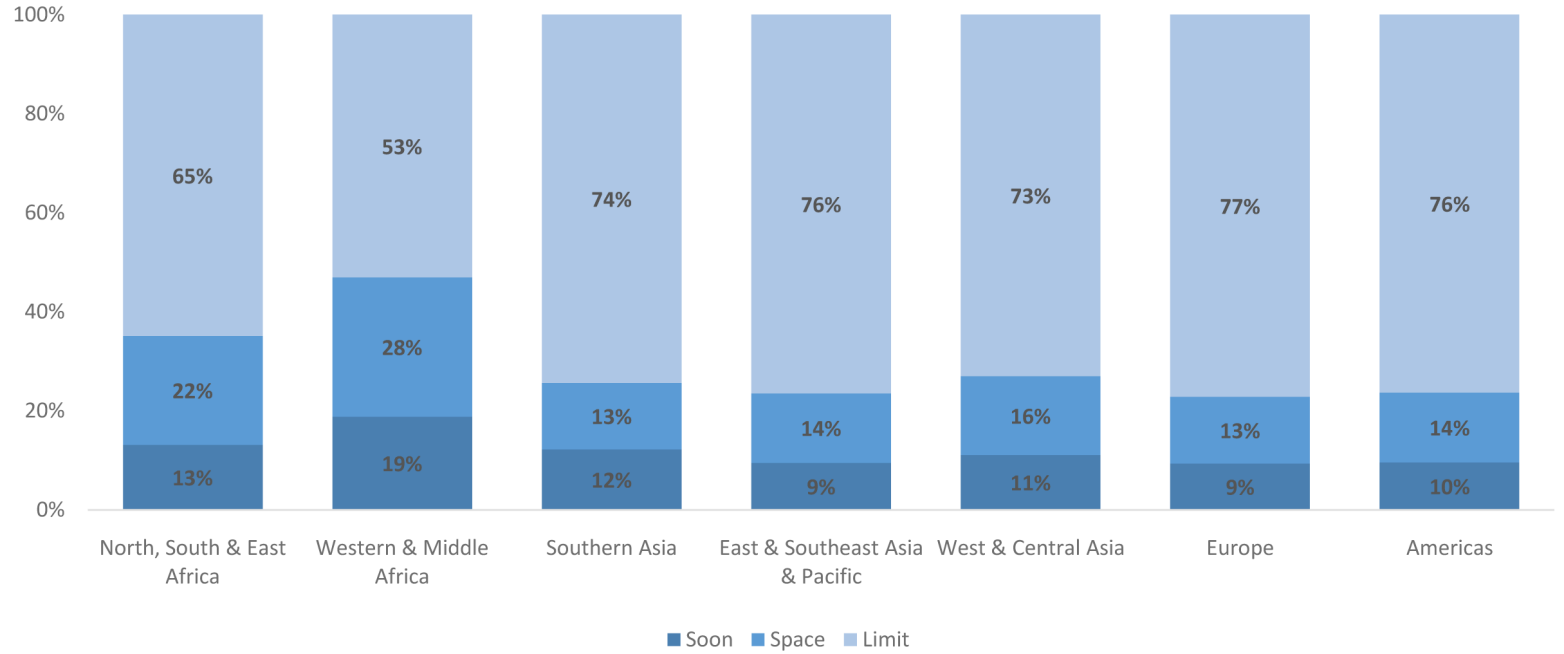
Distribution of married women by market segment and region in 2040. This figure shows the distribution of married women across the three married segments in 2040 by region. In all regions, married women wanting no more children (limit) make up the largest share, though the relative size of this segment varies.

These changes are driven both by changes in the number of married WRA, but also by changes in the distribution of married women across the three segments. The changes in the distribution of women by segment is most pronounced in Western & Middle Africa where the share of married limiters is projected to increase from 38% to 53%. The next largest shift is projected for Northern, Southern & Eastern Africa, where the share of married limiters is projected to increase from 56% to 65%. Outside of Africa the share of married limiters is already above 70% for all remaining regions; only small changes are projected in the relative size of this segment in these regions. See Figure 6 for the distribution of married women by segment in 2040 across regions.

**Figure 7.**
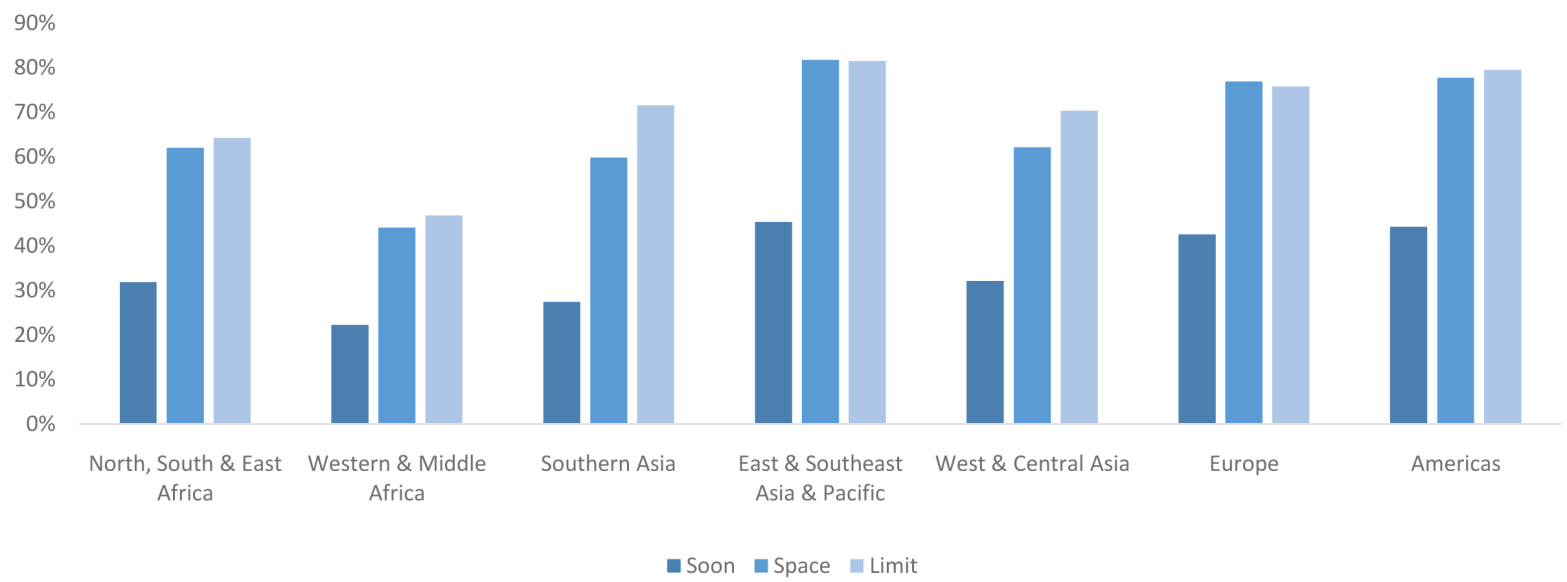
CPR by region for married market segments in 2040. This graph shows the contraceptive prevalence rate (CPR) among each of the three married segments (women waiting a child soon, women wanting to space, and women wanting no more children (limit)) by region. While there is some regional variation in pattern, in general women wanting a child soon are using at about half the level as the other two segments.

***Married users and non-users by segment.*** Globally in 2040, CPR is projected to be 33% among married women who want a child soon, 65% among married spacers, and 73% among married limiters. There is some variation in this pattern by region ( Figure 7); however, in all regions married women who want a child soon are projected to have the lowest CPR among the three groups and represents around half of the CPR of married spacers. 

**Figure 8.**
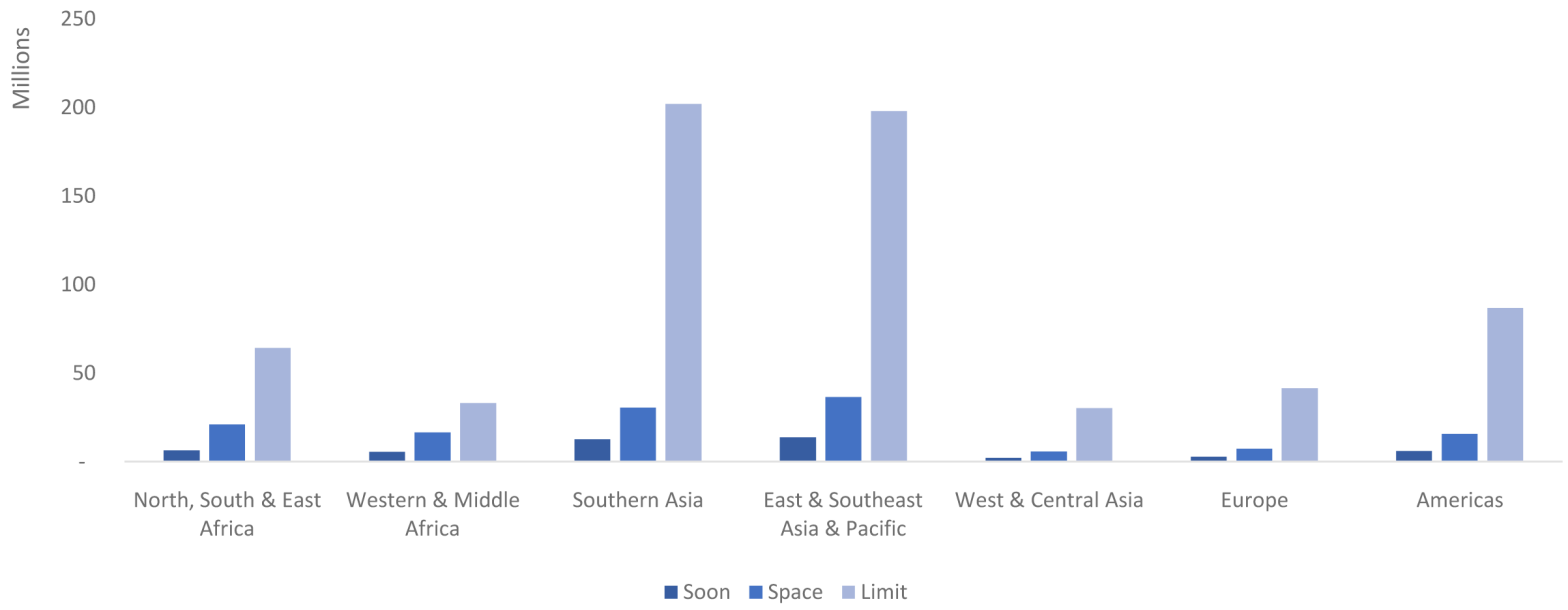
Married users by market segment and region in 2040, in millions. This graph shows the total number of married users (in millions) distributed by market segment and region. Married women wanting no more children (limit) in Southern Asia account for the single largest segment.

In total in 2040 there are a projected 838.4 million married users. The higher CPR among married limiters (in most cases), coupled with the fact that this group is projected to make up more than two-thirds of married WRA in 2040, means that the vast majority of these users will be limiters (78%). Married spacers make up 16% of users, and the remaining 6% of users are married women who want a child soon. The East & Southeast Asia and the Pacific region, which includes China, is projected to be home to the largest number of married users (248 million) followed closely by Southern Asia (245.1 million), which includes India. Married limiters are projected to make up the largest number of users in each region in 2040; however, the magnitude of difference varies across regions ( Figure 8). For example, in Western & Middle Africa 60% of married users are projected to be limiters, while in Southern Asia 82% of married users are projected to be limiters. 

### Unmarried Women

***Unmarried women by segment.*** As seen earlier, the share of women of reproductive age who are married or in union is projected to decline; therefore, the share of unmarried women is projected to increase. Overall, the number of unmarried women is projected to grow from 670 million in 2020 to 859 million in 2040, an increase of 189 million. Increases are projected among both formerly married women (+41.8 million) and never-married women (+147.1 million).

The remainder of the results in this section will focus on the three never-married segments. As noted earlier never-married women are segmented by recency of sex. While the number of never-married women is projected to grow from 546.4 million in 2020 to 693.5 million in 2040, some segments are projected to increase while others are projected to decline. Increases are projected among never-married women who have ever had sex, while the number of never-married women who have never had sex is projected to decline slightly (-6.9m, -2%). The largest increase in both absolute and relative terms is projected among never-married women who have had sex but not recently; this group is projected to increase by 95.7 million (+102%). A large gain is also projected among never-married women who have had sex recently (+58.3 million, +65%).

Patterns of change vary by region as shown in Table 5. The largest gains, in absolute terms, are projected among never-married women who have had recent sex in Africa. By contrast, Southern Asia is projected to see the largest increase in never-married women who have had sex but not recently. Finally, East & Southeast Asia and the Pacific is projected to see the largest decline in the number of never-married women who have never had sex.

**Table 5.** Change in never-married women from 2020 to 2040 by market segment [abs change millions (% change)].

	Recent Sex	Non-recent Sex	No Sex
**North, South & East Africa**	+19.3 (193%)	+12.9 (70%)	+4.8 (16%)
**Western & Middle Africa**	+ 17.6 (270%)	+17.4 (155%)	+9.9 (49%)
**Southern Asia**	+9.6 (2781%)	+20.9 (1792%)	+9.4 (8%)
**East & Southeast Asia & Pacific**	+13.9 (94%)	+17.6 (53%)	-17.9 (-16%)
**Western & Central Asia**	+2.4 (799%)	+5.5 (1555%)	+3.5 (12%)
**Europe**	+23 k (0%)	+4.3 (35%)	-7.5 (-34%)
**Americas**	-4.5 (-13%)	+17.1 (101%)	-9.1 (-22%)
**Total**	**+58.3 (65%)**	**+95.7 (102%)**	**-6.9 (-2%)**

This table shows the change in the number of never-married women in each segment in millions between 2020 and 2040. The number in parentheses indicates the percent change.

**Figure 9.**
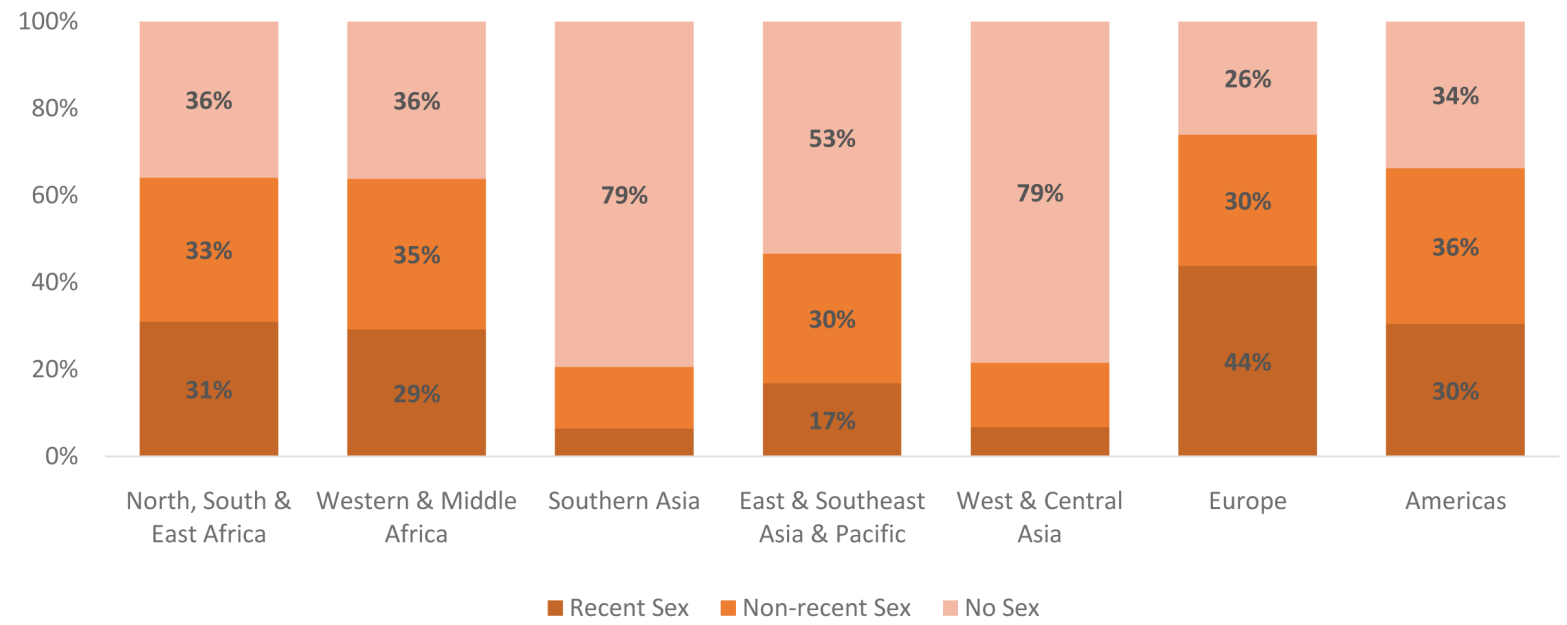
Distribution of never-married women by market segment and region in 2040. This figure shows the distribution of never-married women across the three never-married segments in 2040 by region. There is wide regional variation, especially in the share of never married women who have never had sex.

The changes described are driven both by changes in the number of never-married WRA and shifts in the distribution of never-married women across the three segments. Declines are projected in the share of never-married women who have never had sex in all regions. These declines are projected to be the largest in Southern Asia and Western & Central Asia where pre-marital sex is estimated to be virtually non-existent in 2020. Despite the large projected shift, in both regions nearly 80% of never-married women are still projected to have never had sex. In terms of the share of never-married women who have had recent sex, regions in Africa are projected to have the largest increases. In North, South, & East Africa the share is projected to increase from 17% to 31%, while in Western & Middle Africa the share is projected to increase from 17% to 29%. As seen in Figure 9, there is wide diversity in the distribution of never-married women in 2040 by region, with the most pronounced differences seen in Asia, mirroring similar differences seen today. Despite building in assumptions around norms changes leading to premarital sex, the changes are insufficient to cause a global convergence.

**Figure 10.**
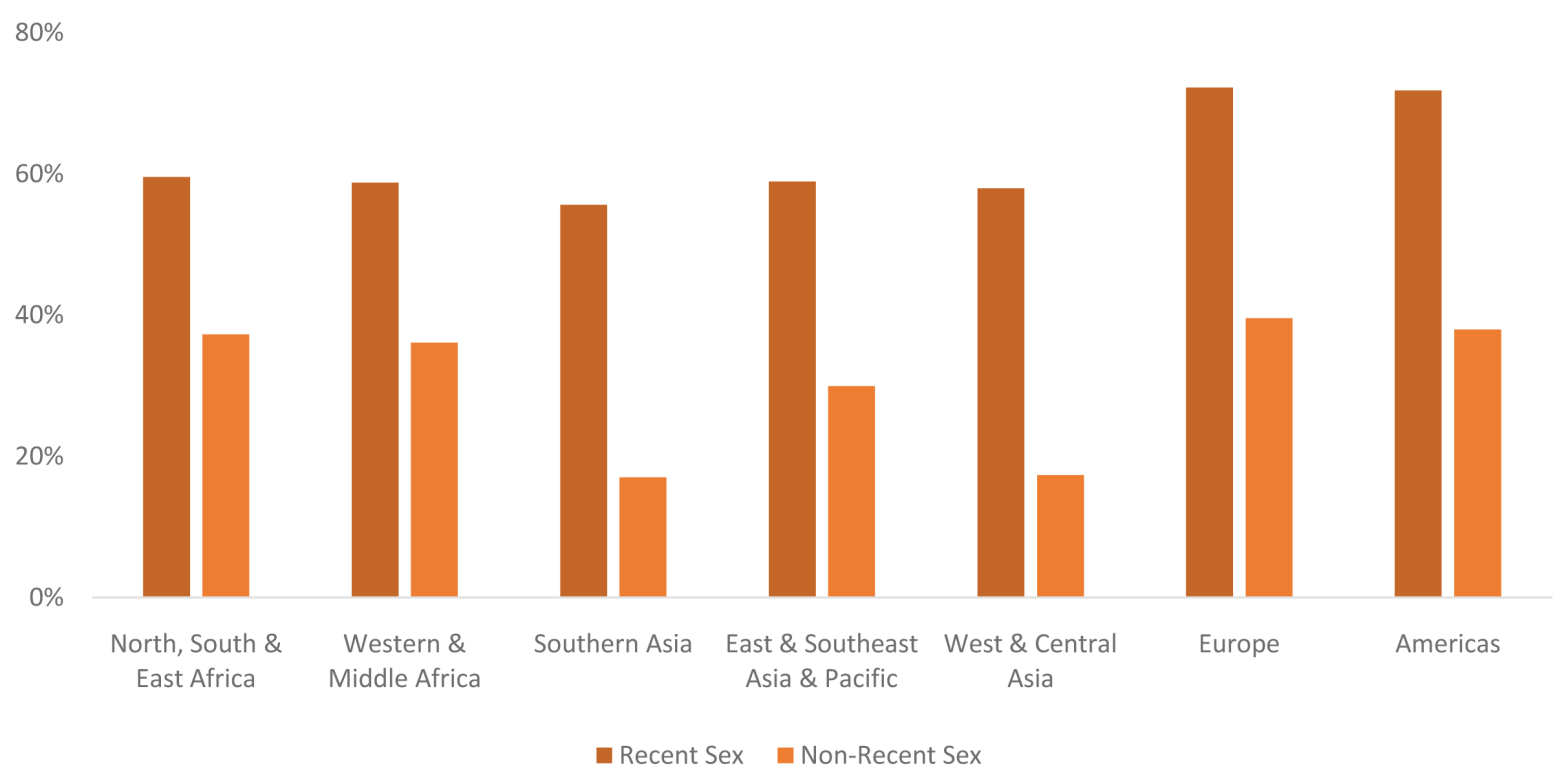
CPR by region for never-married market segments in 2040. This graph shows the contraceptive prevalence rate (CPR) among two of the never-married segments (recent sex and non-recent sex) by region. There is wide regional variation in CPR levels and in the differential between those who had recent sex and non-recent sex.

***Never-married users and non-users by segment.*** Levels of contraceptive use vary by segment. Globally in 2040 the CPR among never-married women who had recent sex is projected to be 63% compared with 32% among never-married women who have had sex but not recently. There is some variation in projected CPR levels by region ( Figure 10); however, in all regions the CPR is projected to be lower among never-married women who have not had sex recently. The largest gap between the two CPRs is seen in Western & Central Asia (58% vs 17%), while the smallest gap is seen in Western & Middle Africa (59% vs 36%), followed closely by Northern, Southern & Eastern Africa (60% vs 37%). 

**Figure 11.**
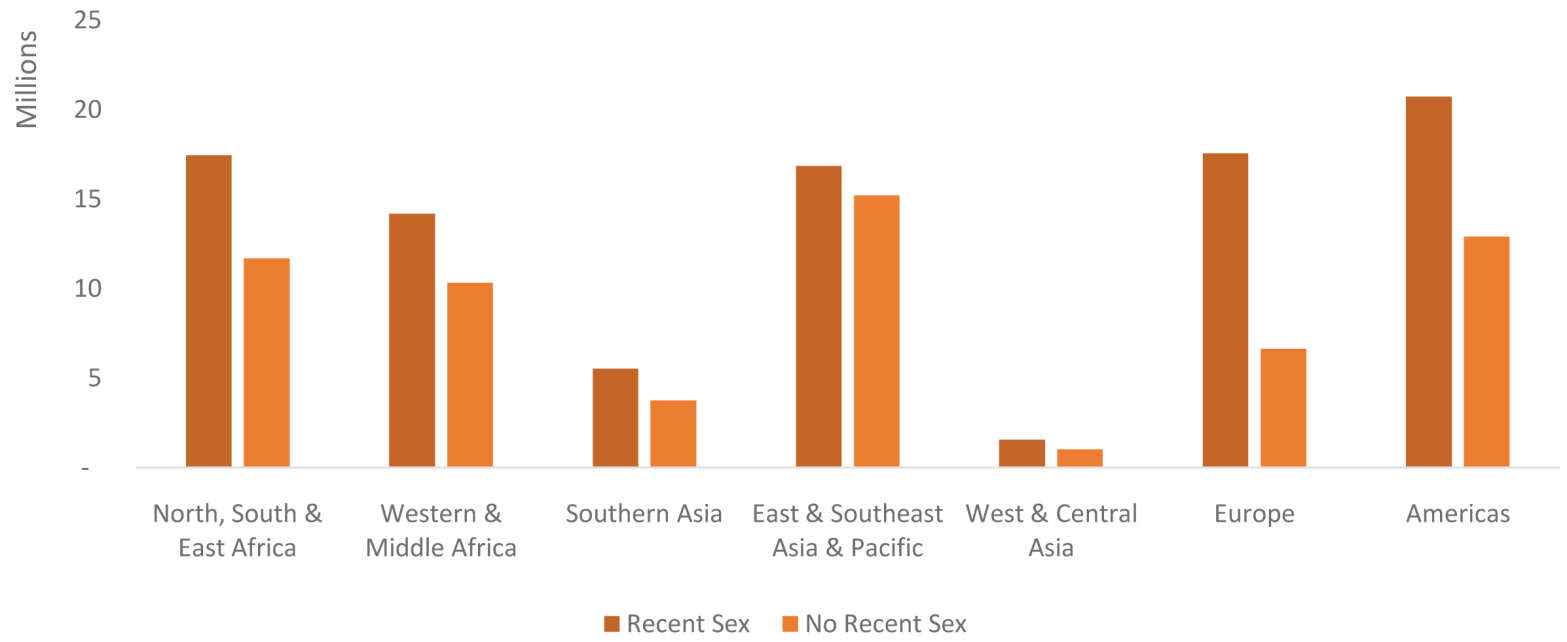
Never-married users by market segment and region in 2040, in millions. This graph shows the total number of never-married users (in millions) distributed by market segment and region. Never married users who have had recent sex in the Americas makes up the single largest segment.

In total there are projected to be 155.4 million never-married users globally in 2040. Nearly two-thirds of these are projected to be women who had recent sex (93.9 million). Across all regions more than half of never-married users are projected to be never-married women who had sex recently. This ranges from only 53% in East & Southeast Asia and the Pacific to 73% in Europe ( Figure 11). In terms of absolute numbers, the Americas is the region projected to have the most never-married users who have had recent sex (20.7 million) while East & Southeast Asia and the Pacific is projected to have the most never-married users who have had sex but not recently (15.2 million). Western & Central Asia is projected to have the fewest users by far in both segments ( Figure 11).

### Married and unmarried women in 2040

Results above have provided detailed projections for married and unmarried women separately. It is also useful to bring these results together to look at a full picture of WRA and contraceptive use across segments. Here, for completeness, results for formerly married women are also included.

**Figure 12.**
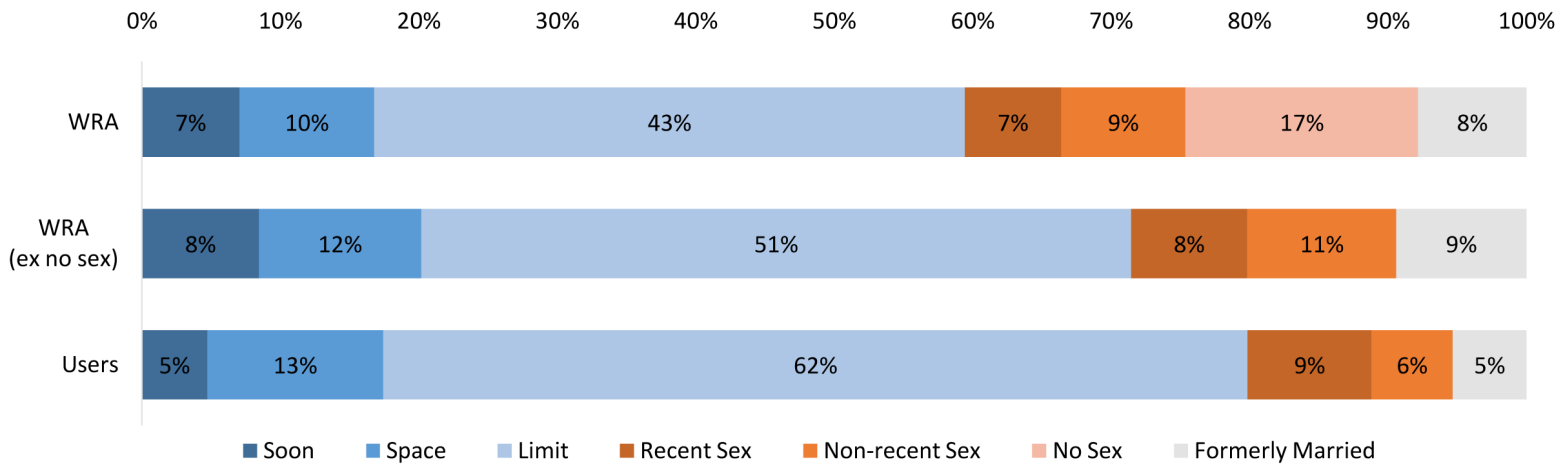
Global distribution of WRA and users by market segment in 2040. This graph shows the global distribution of women of reproductive age (WRA) and users by market segment in 2040. WRA are shown with the never married never had sex (‘no sex’) segment removed to allow easier compatibility with the distribution of users.

Married limiters are projected to make up the largest segment of both WRA (43%) and users (62%). The next largest segment of WRA is projected to be never-married women who have never had sex (17%). The remaining WRA are projected to be fairly evenly distributed among the remaining segments. In terms of users, the next largest segment is projected to be married spacers (13%) followed by never-married women who have had recent sex (9%). The remaining three segments each are projected to have a similar number of users ( Figure 12).

**Figure 13.**
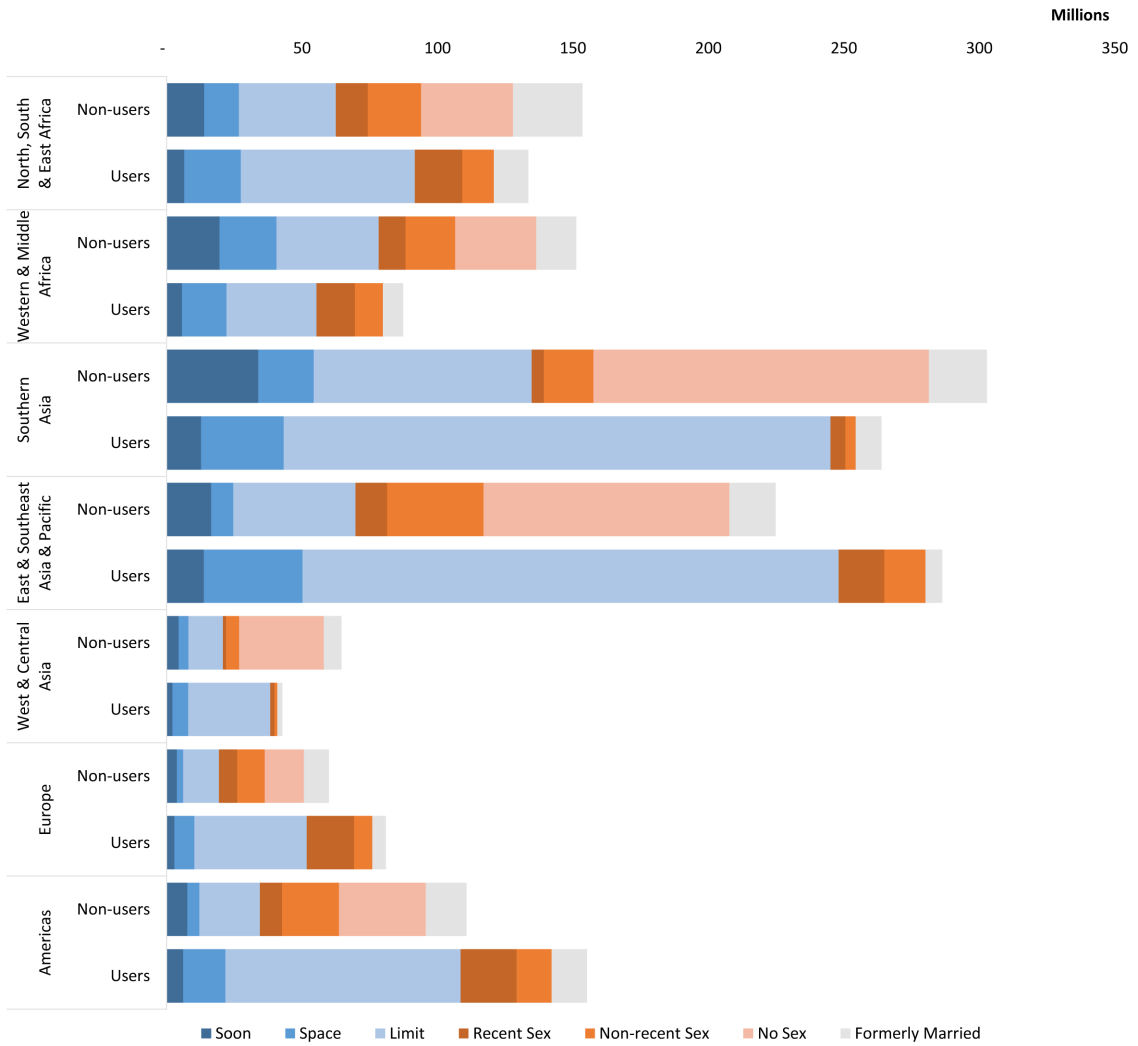
WRA by use, region and market segment in 2040, in millions. This graph shows all women of reproductive age (WRA) in 2040 distributed by region, market segment, and contraceptive use and non-use. Each region shows 2 stacked bars, the first shows non-users by market segment and the second shows users by market segment.

Regional differences will persist in both the size and relative shares of users and non-users by segment ( Figure 13). Among users, married limiters are projected be the single largest segment in all regions in 2040; however, the relative size of their role will vary widely from 38% of users in Western & Middle Africa to 76% in Southern Asia. Married spacers are projected to make up the next largest segment of users in most regions, with the exception of Europe and the Americas where never-married women who have had recent sex are projected to be the next largest segment. 

Among non-users, both married limiters and never-married women who have never had sex are projected to hold the largest shares in 2040, though in varying proportions. In the three Asian regions, more than 40% of non-users are projected to be comprised of never-married women who have never had sex. In the remaining regions, there is projected to be a more even split between this group and limiters.

Turning to absolute numbers of users and non-users, distinct patterns emerge. Southern Asia is projected to have the largest number of non-users in 2040 (302.8 million), while East & Southeast Asia and the Pacific is projected to be the home to the most users (286.4 million) although followed closely by Southern Asia (263.9 million). While these two regions are projected to be home to the largest number of users and non-users for most segments, there are some exceptions. For example, the largest numbers of never-married users who have had recent sex are projected to be found in the Americas (20.7 million), followed by Europe (17.6 million) and North, South, & East Africa (17.5 million). While Southern Asia is projected to be home to a quarter of all users in 2040, only 6% of both never-married user segments are projected to reside in the region. 

## Discussion

The evolution of the global family planning market will be driven by the following forces: demographic changes, evolving fertility preferences, shifts in social norms, increased utilization of contraceptives, and other social and economic changes likely to impact on method preferences. In terms of demographic changes, in Africa and much of Asia (Southern Asia and West & Central Asia) there will continue to be large demographic momentum leading to increased numbers of women of reproductive age and therefore larger markets of potential users. By contrast, in parts of Asia (especially China), Europe, the Americas, and the Pacific the numbers of women of reproductive age will stagnate or decline. Evolutions in fertility preferences are projected to be most pronounced in places with current preferences for large families, where a decline in ideal family size is expected. In some places this decline will be rapid, in others it will be slower. As these changes happen, so too will there be changes in how women desire to space and time their pregnancies. Changes will be most notable in Africa, where a shift to smaller families will mean more women spending more of their reproductive years trying to avoid any further pregnancies, thus growing the market among married limiters. Perhaps most speculatively, there will be changes in patterns of marriage and in sexual behaviors outside marriage. Although there are some exceptions, most countries will see delays in marriages leading to more women spending a larger share of their reproductive years unmarried. This, coupled with liberalization of social norms leading to more premarital sexual activity, will expand the market of contraceptive among never-married women. Next, the use of contraceptives among both married and never-married women is expected to increase. These changes are most concentrated in those areas where use is the lowest today – namely in Africa and parts of Asia. New CTs will play a role in driving these projected changes and could potentially lead to even larger gains by addressing barriers to uptake related to today’s methods. However, the market of non-users must be examined carefully, as the needs and therefore likelihood of adoption of contraception varies widely among non-users. For example, some married non-users who desire no more children may have low need for contraceptives due to infrequent sex or being post-menopausal. Finally, other changes, not directly accounted for in this work, are also expected. Economic and social development, urbanization, and cultural shifts that may lead women to be more empowered in controlling their reproductive choices are likely. These, in turn, will impact on the types of CTs that the women of 2040 are likely to desire. 

The results presented in this paper—WRA, users, and non-users by segment—provide the starting point for understanding the future market for a new CT. Future research by these authors will address how new CTs can best align with the evolving contraceptive market and which women will be most likely to switch to or adopt a new technology based on their needs and preferences. 

### Market evolution by region

The driving forces of change vary widely by region, meaning distinct narratives emerge about the projected market changes ahead. Africa is projected to see the most change. Rapid population growth will continue, fertility preferences will change as family size norms change, and in most places the age at marriage will increase. The largest growth in this region is projected to be among married limiters, suggesting a need for expanding access to longer-duration CTs that can best meet this segment’s needs. In contrast to much of Asia, tubal ligation has never captured a significant share of the family planning market in Africa, suggesting a role for a new technology that can provide more acceptable long-term alternative. However, it is important to note that despite this increase, married limiters are projected to account for less than half of users in 2040, meaning a diverse set of contraceptive needs will exist across the region. For example, by 2040, it is projected that there will be a large number of never-married users, as well as sexually active never-married non-users. Large shares of these never-married women are projected to be having infrequent sex, a factor that is likely to impact their contraceptive needs. 

Asia is a complicated and diverse market. Even though most countries are near or approaching replacement level fertility, population growth continues. In Southern Asia, there are projected to be increasing numbers of both married and never-married women of reproductive age. On the other hand, in Eastern Asia, the number of married women is likely to decline and the number of never-married WRA will increase. Among the married women, there is projected to be a continued trend toward a larger percent being limiters as ideal family sizes are potentially achieved earlier in their reproductive lives. Less predictable, but likely, a larger percent of never-married women will be sexually active. While still a small share, given the sheer number of never-married women this translates more than one hundred million women in 2040. The contraceptive needs of these never-married women will be distinct from the needs of married limiters who dominate so much of use today. 

Europe and the Americas are for the most part markets that are projected to stagnate or decline. This is due to demographic forces; for decades fertility rates have been at or below replacement level for most countries in these regions. In Europe, despite an overall decline in WRA and declines in most segments, an increase is projected among never-married women who have had sex. An interesting phenomenon in the Americas is that married limiters and women who want a child soon are projected to gain shares among the married women. The classic category of “spacers” is projected to shrink as a relatively small fraction of a woman’s sexually active years is spent between births. This all points to diverse and varying contraceptive needs within these markets.

### Limitations

The largest limitation of this work is that the future is uncertain, especially when trying to project two decades into the future. There are many unpredictable changes that this modeling is not able to account for which could influence not only the trajectories of the seven segments, but also the contraceptive needs and preferences of women within each segment. Demographic changes are perhaps most certain; the momentum behind these changes is often slow to change and methodological developments in projection models are able to capture expected shifts. Projecting large scale changes in social norms, most notably how changes in sexual activity before marriage may unfold, is perhaps the most challenging. 

Further, data availability varies by country and region. Collectively DHS and MICS surveys provide good coverage for developing countries; data for high-income countries is more sparse and less standardized. However, given high contraceptive use and low fertility in many high-income countries, there is less room for variation in many of the key model assumptions, minimizing the impact of more limited data.

Sensitivity testing will be done around key assumptions to quantify the potential impact of the uncertainty in the modelling. 

### Conclusion

This analysis projects the impact of demographic changes, evolving fertility preferences, shifts in sexual activity outside of marriage and increased utilization of contraceptives in shaping the contraceptive market of 2040. Results show that there is not one global market, but distinct markets that vary in size and shape across the world. This diversity suggests that a range of different new CTs could have potential for uptake. The segments described in this paper are only a first step to understanding future contraceptive needs. Subsequent work will build in new data that captures how contraceptive preferences vary by segment in order to understand which new methods are most likely to be used by which women. 

Uptake alone, however, should not be the only metric by which the impact of a new CT is judged. To understand the public health impact of a new CT it is important not only to account for its uptake, but also the change in the risk of unintended pregnancy among those who may use the method. A method that appeals more widely to women who would otherwise use no method, traditional methods, or less effective modern methods will have a larger benefit in reducing women’s risk of unintended pregnancy. The modeling described in this paper was developed with this end in mind, and the validation of meaningful differences in contraceptive use by segment shows promise for subsequent work to meaningfully project future preferences for new CTs and ultimately the impact of those CTs in terms of unintended pregnancies averted. By accounting not only for future market size for a new CT, but also the potential health impact, research and development (R&D) investments can be targeted towards those methods most likely to reduce the burden of unintended pregnancy. 

## Data availability

### Source data

**DHS StatCompiler**

Analysis of fertility preferences by TFR took relevant indicators from the DHS’s StatCompiler tool for the following surveys: Burundi (2016-17, 2010, 1987), Comoros (2012, 1996), Eritrea (2002, 1995), Ethiopia (2016, 2011, 2005, 2000), Kenya (2014, 2008-09, 2003, 1998, 1993, 1989), Madagascar (2008-09, 2003-04, 1997, 1992), Malawi (2015-16, 2010, 2004, 2000, 1992), Mozambique (2015, 2011, 2003, 1997), Rwanda (2014-15, 2010, 2007-08, 2005, 2000, 1992), Tanzania (2015-16, 2010, 2004-05, 1999, 1996, 1991-92), Uganda (2016, 2011, 2006, 2000-01, 1995, 1988-89), Zambia (2013-14, 2007, 2001-02, 1996, 1992), Zimbabwe (2015, 2010-11, 2005-06, 1999, 1994, 1988), Angola (2015-16), Cameroon (2011, 2004, 1998, 1991), Central African Republic (1994-95), Chad (2014-15, 2004, 1996-97), Congo (2011-12, 2005), Congo Democratic Republic (2013-14, 2007), Gabon (2012, 2000), Sao Tome and Principe (2008-09), Egypt (2014, 2008, 2005, 2003, 2000, 1995, 1992, 1988), Morocco (2003-04, 1992, 1987), Sudan (1989-90), Tunisia (1988), Botswana (1988), Eswatini (2006-07), Lesotho (2014, 2009, 2004), Namibia (2013, 2006-07, 2000, 1992), South Africa (2016, 1998), Benin (2017-18, 2011-12, 2006, 2001, 1996), Burkina Faso (2010, 2003, 1998-99, 1993), Cote d'Ivoire (2011-12, 1998-99, 1994), Gambia (2013), Ghana (2014, 2008, 2003, 1998, 1993, 1988), Guinea (2012, 2005, 1999), Liberia (2013, 2007, 1986), Mali (2012-13, 2006, 2001, 1995-96, 1987), Mauritania (2000-01), Niger (2012, 2006, 1998, 1992), Nigeria (2013, 2008, 2003, 1990), Senegal (2017, 2016, 2015, 2014, 2012-13, 2010-11, 2005, 1997, 1992-93, 1986), Sierra Leone (2013, 2008), Togo (2013-14, 1998, 1988), Kazakhstan (1999, 1995), Kyrgyz Republic (2012, 1997), Tajikistan (2017, 2012), Turkmenistan (2000), Uzbekistan (1996), Cambodia (2014, 2010, 2005, 2000), Indonesia (2012, 2007, 2002-03, 1997, 1994, 1991, 1987), Myanmar (2015-16), Philippines (2017, 2013, 2008, 2003, 1998, 1993), Thailand (1987), Timor-Leste (2016, 2009-10), Vietnam (2002, 1997), Afghanistan (2015), Bangladesh (2014, 2011, 2007, 2004, 1999-00, 1996-97, 1993-94), India (2015-16, 2005-06, 1998-99, 1992-93), Maldives (2016-17, 2009), Nepal (2016, 2011, 2006, 2001, 1996), Pakistan (2017-18, 2012-13, 2006-07, 1990-91), Sri Lanka (1987), Armenia (2015-16, 2010, 2005, 2000), Azerbaijan (2006), Jordan (2017-18, 2012, 2009, 2007, 2002, 1997, 1990), Turkey (2003, 1998, 1993), Yemen (2013, 1997, 1991-92), Moldova (2005), Ukraine (2007), Albania (2017-18, 2008-09), Dominican Republic (2013, 2007, 2002, 1999, 1996, 1991, 1986), Haiti (2016-17, 2012, 2005-06, 2000, 1994-95), Trinidad and Tobago (1987), El Salvador (1985), Guatemala (2014-15, 1998-99, 1995, 1987), Honduras (2011-12, 2005-06), Mexico (1987), Nicaragua (2001, 1998), Bolivia (2008, 2003, 1998, 1994, 1989), Brazil (1996, 1986), Colombia (2015, 2010, 2005, 2000, 1995, 1990, 1986), Ecuador (1987), Guyana (2009), Paraguay (1990), Peru (2012, 2011, 2010, 2009, 2007-08, 2004-06, 2000, 1996, 1991-92, 1986).

StatCompiler is available online at: https://www.statcompiler.com/en/

**DHS secondary analysis**

Data used in this study are from the Individual Women’s Recode datasets of the following DHS surveys: Afghanistan (2015), Albania (2008-09), Angola (20015-16), Armenia (20015-16), Azerbaijan (2006), Bangladesh (2014), Benin (2017-18), Bolivia (2008), Brazil (1996), Burkina Faso (2010), Burundi (2016-17), Cambodia (2014), Central African Republic (1994-95), Chad (2014-15), Columbia (2015), Comoros (2012), Congo (2011-12), Cote d’Ivoire (2011-12), Dominican Republic (2013), DRC (2013-14), Egypt (2014), Eswatini (2006-07), Ethiopia (2016), Gabon (2012), Gambia (2013), Ghana (2014), Guatemala (2014-15), Guinea (2018), Guyana (2009), Haiti (2017), Honduras (2011-12), India (2015-16), Indonesia (2017), Jordan (2017-18), Kazakhstan (1999), Kenya (2014), Kyrgyz Republic (2012), Lesotho (2014), Liberia (2013), Madagascar (2008-09), Malawi (2015-16), Maldives (2016-17), Mali (2018), Morocco (2003-04), Mozambique (2011), Myanmar (2015-16), Namibia (2013), Nepal (2016), Nicaragua (2001), Niger (2012), Nigeria (2018), Pakistan (2017-18), Peru (2012), Philippines (2017), Rwanda (2014-15), Sao Tome and Principe (2008-09), Senegal (2017), Sierra Leone (2013), South Africa (2016), Tajikistan (2017), Tanzania (2015-16), Timor Leste (2016), Togo (2013-14), Turkey (2003), Uganda (2016), Yemen (2013), Zambia (2018), Zimbabwe (2015).

Datasets are available from the Demographic and Health Survey (DHS) website. Access to the datasets requires registration and is granted only for legitimate research purposes. A guide for how to apply for dataset access is available at: https://dhsprogram.com/data/Access-Instructions.cfm.

**MICS secondary analysis**

Data used in this study are from the Women in Reproductive Age datasets of the following surveys: Barbados (2012), Belarus (2012), Belize (2011), Bhutan (2010), Bosnia and Herzegovina (2011), Cameroon (2014), CAR (1994-95), Costa Rica (2011), Cuba (2014), Dominican Republic (2014), El Salvador (2014), Guyana (2009), Kazakhstan (1999), Kyrgyz Republic (2012), Mexico (2015), Moldova (2012), Mongolia (2013-4), Montenegro (2013), Panama (2013), Republic of Moldova (2012), Serbia (2014), South Sudan (2010), Swaziland (2006-07), TFYR Macedonia (2011), Thailand (2012), Ukraine (2012), Uzbekistan (1996), Vietnam (2002).

Datasets are available from the Multiple Indicator Cluster (MICS) website. Access to the datasets requires registration and is granted only for legitimate research purposes. A guide for how to apply for dataset access is available at: https://mics.unicef.org/visitors/sign-up.

**Marital status data**

Data on marital status was taken from the UN’s World Marriage Data 2017 database and used the most recently available source for the following countries: United States, Australia, Austria, Azerbaijan, Bangladesh, Belgium, Botswana, Brunei Darussalam, Bulgaria, Canada, Chile, China, China, Hong Kong SAR, China, Macao SAR, Croatia, Curaçao, Cyprus, Czechia, Denmark, Djibouti, Equatorial Guinea, Estonia, Finland, France, French Polynesia, Georgia, Germany, Greece, Hungary, Iceland, Iran (Islamic Republic of), Iraq, Ireland, Israel, Italy, Japan, Jordan, Kuwait, Lao People's Democratic Republic, Latvia, Lithuania, Luxembourg, Malta, Mauritius, Mayotte, Netherlands, New Caledonia, Norway, Papua New Guinea, Poland, Portugal, Republic of Korea, Romania, Saint Vincent and the Grenadines, Serbia, Slovenia, Spain, Sri Lanka, Suriname, Sweden, Switzerland, Tonga, Trinidad and Tobago, Turkey, Uruguay, Venezuela (Bolivarian Republic of), Viet Nam.

The database can be downloaded at: https://www.un.org/development/desa/pd/data/world-marriage-data

## References

[ref-1] Biddlecom A Riley T Darroch JE : Future Scenarios of Adolescent Contraceptive Use Cost and Impact in Developing Regions.New York: Guttmacher Institute.2018. Reference Source

[ref-3] Calliope: The Contraceptive Pipeline Database. 2020; Accessed 3/16/2020. Reference Source

[ref-5] Darroch JE Sedgh G Ball H : Contraceptive Technologies: Responding to Women’s Needs.New York: Guttmacher Institute,2011. Reference Source

[ref-7] FHI360: User Perspectives on New Long-acting Contraceptive Technologies- Final Report, North Caroline: FHI360. 2017. Reference Source

[ref-8] Guttmacher Institute: Adding It Up: The Costs and Benefits of Investing in Sexual and Reproductive Health 2017.New York: Guttmacher Institute.2017. Reference Source 15352318

[ref-9] ICF 2004-2019: Demographic and Health Surveys (various) [Datasets].Funded by USAID. Rockville, Maryland: ICF [Distributor]. Reference Source

[ref-10] ICF: The DHS Program STATcompiler.Funded by USAID.2012. Reference Source

[ref-11] Institute of Medicine (US) Committee on New Frontiers in Contraceptive Research: New Frontiers in Contraceptive Research: Chapter 4 Improving Contraceptive Use and Acceptability.Washington DC.2004. Reference Source

[ref-12] Jain A Winfrey W : Contribution of Contraceptive Discontinuation to Unintended Births in 36 Developing Countries. *Stud Fam Plann.* 2017;48(3):269–278. 10.1111/sifp.12023 28398595

[ref-14] UNICEF: Multiple indicator cluster surveys [Datasets].2019. Reference Source

[ref-15] United Nations, Department of Economic and Social Affairs, Population Division: World Marriage Data 2017 (POP/DB/Marr/Rev2017).2017. Reference Source

[ref-16] United Nations, Department of Economic and Social Affairs, Population Division: World Population Prospects 2019.New York: United Nations,2019a. Reference Source

[ref-17] United Nations, Department of Economic and Social Affairs, Population Division: Estimates and Projections of Family Planning Indicators 2019.New York: United Nations,2019b. Reference Source

[ref-18] Weinberger M Miller N Skibiak J : Commodity gap analysis 2019.RHSC,2019. Reference Source

