# Supplementary material for: Quantifying the potential market for new contraceptive technologies: global projections of 2040 contraceptive needs and preferences
Source: Gates Open Res. 2021 Oct 22;5:152. [Version 1] doi: 10.12688/gatesopenres.13400.1 (PMC8649629; doi:10.12688/gatesopenres.13400.1)
Supplement: Peer review history

# Peer review: Reviewer response for version 1

**DOI:** 10.21956/gatesopenres.14652.r31400

**Published:** 2021-12-06

**Authors:** Kate H. Rademacher

**Affiliations:** 1 FHI 360, Durham, NC, USA

This an excellent article and timely analysis to help inform investments in new contraceptive research and development (R&D) as well as broader introduction of lesser-used family planning methods. The authors do an excellent job of clearly describing their methods and results, while recognizing the uncertainties related to predicting future trends. In particular, the finding that married limiters are projected to make up the largest segment of both WRA and users is interesting and important.

 I wanted to share several observations and suggestions for consideration:

1. Although the paper focuses on implications for development of new contraceptive technologies, the paper would be strengthened by also acknowledging the potential role of expanding access to existing family planning methods that are currently underutilized, including those that might appeal to married limiters. This could include reversible long-acting methods (e.g., the hormonal IUD) or permanent methods (e.g., vasectomy). While regional differences in uptake of tubal ligation is briefly mentioned, other existing methods are not mentioned. In addition to resources in R&D, investments are needed to increase access to highly effective methods that are currently not available to all populations. 
2. One of the seven market segments was defined as, “Formerly married women.” However, it was unclear what assumptions were made about the sexual activity of this group. This seems like it could actually be a large market segment, but little was said about this group in the paper. Suggest expanding here.

Several minor corrections are also recommended:

**1. **For this citation: “FHI 360: User Perspectives on New Long-acting Contraceptive Technologies- Final Report, North Carolina.” Please note that several articles have been published in the peer-reviewed literature with findings based on this assessment since the original report came out. I suggest updating this citation. For example, see the following:

- Callahan *et al.* (2019 ^1^).
- Brunie *et al.* (2019 ^2^).
- Cartwright *et al.* (2020 ^3^).

There is also other recent related research by Callahan, Brunie and colleagues that could be cited.

**2.** For this statement: “Large global initiatives are making investments in developing new technologies (for example, the BMGF CTI Exchange and the USAID-funded Envison FP project ^1^)”. To clarify, the CTI Exchange website is a knowledge sharing platform. I believe you meant to mention the “Contraceptive Technology Innovation Initiative," which is the Gates-funded project that is supporting contraceptive R&D. See: https://www.fhi360.org/projects/contraceptive-technology-innovation-cti-initiative

 Also, a new USAID project has been awarded; this is called Innovate FP that could be mentioned as well as Envision FP. See: https://www.fhi360.org/projects/innovate-fp-expanding-options-enhancing-method-choice

 Thank you again for publishing this excellent, insightful article! It is an important contribution to the literature.

Is the work clearly and accurately presented and does it cite the current literature?

Yes

If applicable, is the statistical analysis and its interpretation appropriate?

I cannot comment. A qualified statistician is required.

Are all the source data underlying the results available to ensure full reproducibility?

Yes

Is the study design appropriate and is the work technically sound?

Yes

Are the conclusions drawn adequately supported by the results?

Yes

Are sufficient details of methods and analysis provided to allow replication by others?

Yes

Reviewer Expertise:

Family planning, long-acting reversible contraception, new method introduction

I confirm that I have read this submission and believe that I have an appropriate level of expertise to confirm that it is of an acceptable scientific standard.

## References

[1] : Potential user interest in new long-acting contraceptives: Results from a mixed methods study in Burkina Faso and Uganda. *PLoS One* .2019;14(5) : 10.1371/journal.pone.0217333 e0217333 10.1371/journal.pone.0217333 31136612PMC6538161

[2] : Developing acceptable contraceptive methods: Mixed-method findings on preferred method characteristics from Burkina Faso and Uganda. *Gates Open Res* .2019;3: 10.12688/gatesopenres.12953.2 1205 10.12688/gatesopenres.12953.2 31984310PMC6963344

[3] : What About Methods for Men? A Qualitative Analysis of Attitudes Toward Male Contraception in Burkina Faso and Uganda. *International Perspectives on Sexual and Reproductive Health* .2020;46: 10.1363/46e9720 10.1363/46e9720 PMC778015532985988

# Peer review: Reviewer response for version 1

**DOI:** 10.21956/gatesopenres.14652.r31271

**Published:** 2021-11-11

**Authors:** Bamikale Feyisetan

**Affiliations:** 1 Global Health Bureau, Office of Population and Reproductive Health, United States Agency for International Development (USAID), Washington, DC, USA

This analysis shows what the 2040 contraceptive market would look like based on assumed demographic changes, evolving fertility preferences, shifts in sexual activity outside of marriage and increased utilization of contraceptives. Using the United Nation’s (UN) population projections of the number of women of reproductive age (WRA) as the basis of their work, seven market segments were defined from observed and predicted compositions of the women by marital status, fertility intentions, and patterns of sexual activity outside of marriage. Each market segment is further subdivided by contraceptive use status – use versus non-use. Market segmentation is based on UN projections, household surveys, census data, and modeling techniques.

 This is a comprehensive analysis that uses appropriate input estimations and projections to identify the potential sizes and regional patterns of future (2040) contraceptive markets. According to the authors, the largest market increases will be seen in Africa, particularly among the segment of married women wanting no more children. By contrast, Asia will see declines across all three married segments, coupled with increases among sexually active unmarried segments. Levels of contraceptive use are projected to vary widely by segment, with differential patterns across regions. The analysis further shows that there is not one global market, but distinct markets that vary in size and shape across the world. This diversity suggests that a range of different new CTs could have potential for uptake. These findings are important for informing planning for future contraceptive needs at global, regional and country levels.

**Issues to note/address:**

- The market segmentation process might be a bit difficult to follow for many readers. It might be helpful to have an appendix that provides a sequential step-by-step guide on how estimates of the input indicators were obtained and applied to the country’s projected populations to obtain the market size estimates.
- Since the market size estimates were developed by country, an appendix that shows the market size estimate for each country would be useful to country-level FP program planners.
- A big question that remains unanswered is “what methods of contraception would be dominant among future contraceptive users the majority of whom would be married limiters?" Maybe the next paper/analysis will address answer this question.
- A major component of the analysis for married women is obtaining the global convergence point for the share of married women spacing or limiting at replacement fertility level. Since reaching the replacement level implies declines in TFR over time, it might make sense to reverse the TFR scale in figures 1 and 2 – from higher to lower values. That way, figures 1 and 2 will more appropriately show what happens to the proportions/share of married women that are spacers and limiters, respectively, as TFR declines. While this would affect the values in Table 2, it won’t affect the conclusions drawn from the analysis.
- It should be clearly stated in the paper that the market sizes for married women were obtained by applying to the projected 2040 TFR the regression coefficients from the linear progression of each country’s latest share of married women by fertility preferences to the global convergence point at a replacement fertility level.
- I am happy to note that the authors recognized that projecting populations over two decades is more prone to uncertainties and hence might be less accurate than projections over say, five or ten years. The inaccuracies/uncertainties pertain not only to projected populations but also to the projected behavioral practices – fertility intentions, sexual behavior and contraceptive use. I would like to suggest that the authors make this point early in the paper. Doing so does not take away from the value of the paper.
- The authors might want to state how well the linear projections of the populations between 2030 and 2040 align with the UNDP projections that end in 2030.

Is the work clearly and accurately presented and does it cite the current literature?

Yes

If applicable, is the statistical analysis and its interpretation appropriate?

Yes

Are all the source data underlying the results available to ensure full reproducibility?

Yes

Is the study design appropriate and is the work technically sound?

Yes

Are the conclusions drawn adequately supported by the results?

Yes

Are sufficient details of methods and analysis provided to allow replication by others?

Yes

Reviewer Expertise:

Population and Reproductive Health

I confirm that I have read this submission and believe that I have an appropriate level of expertise to confirm that it is of an acceptable scientific standard.
